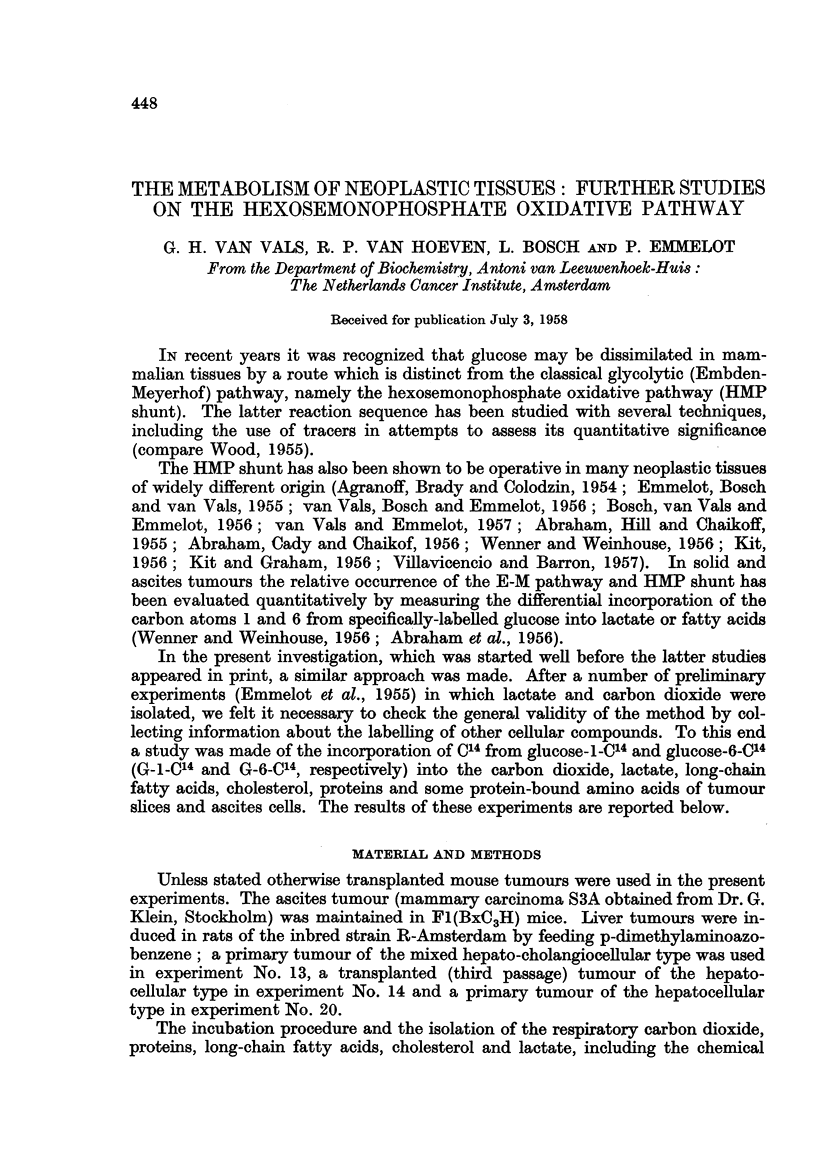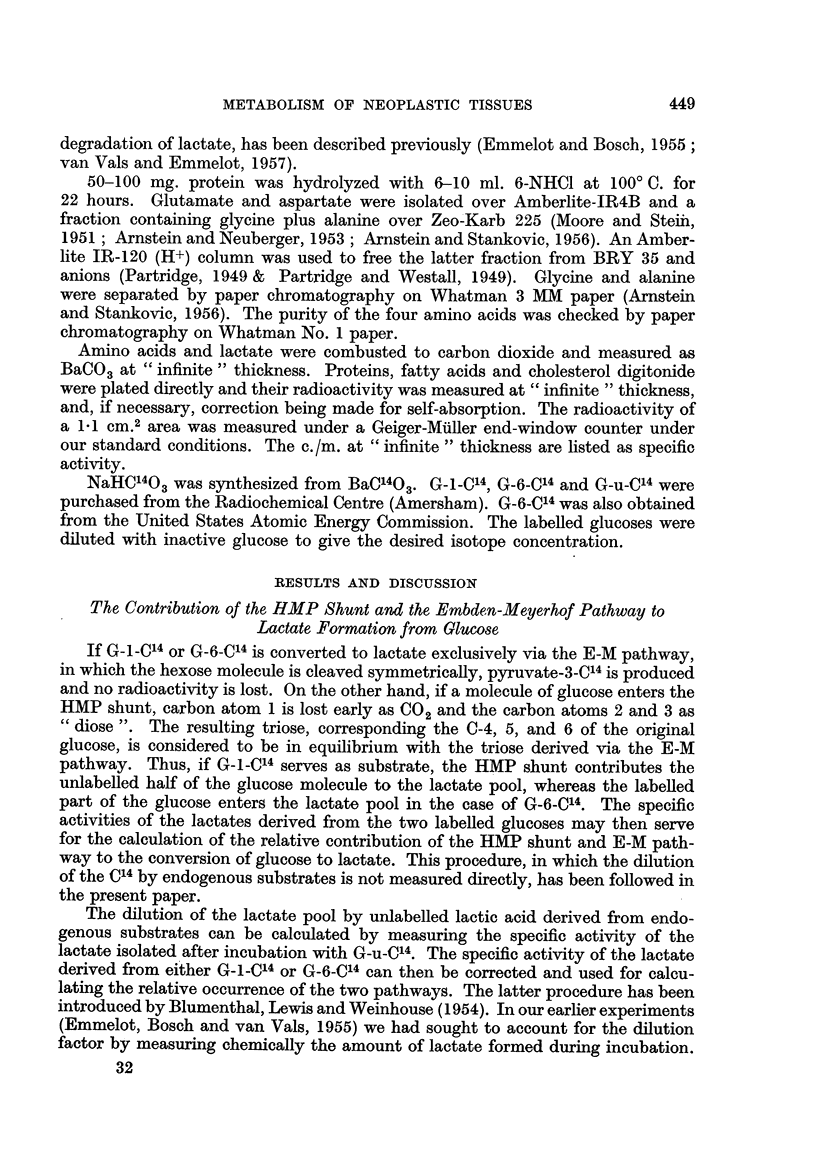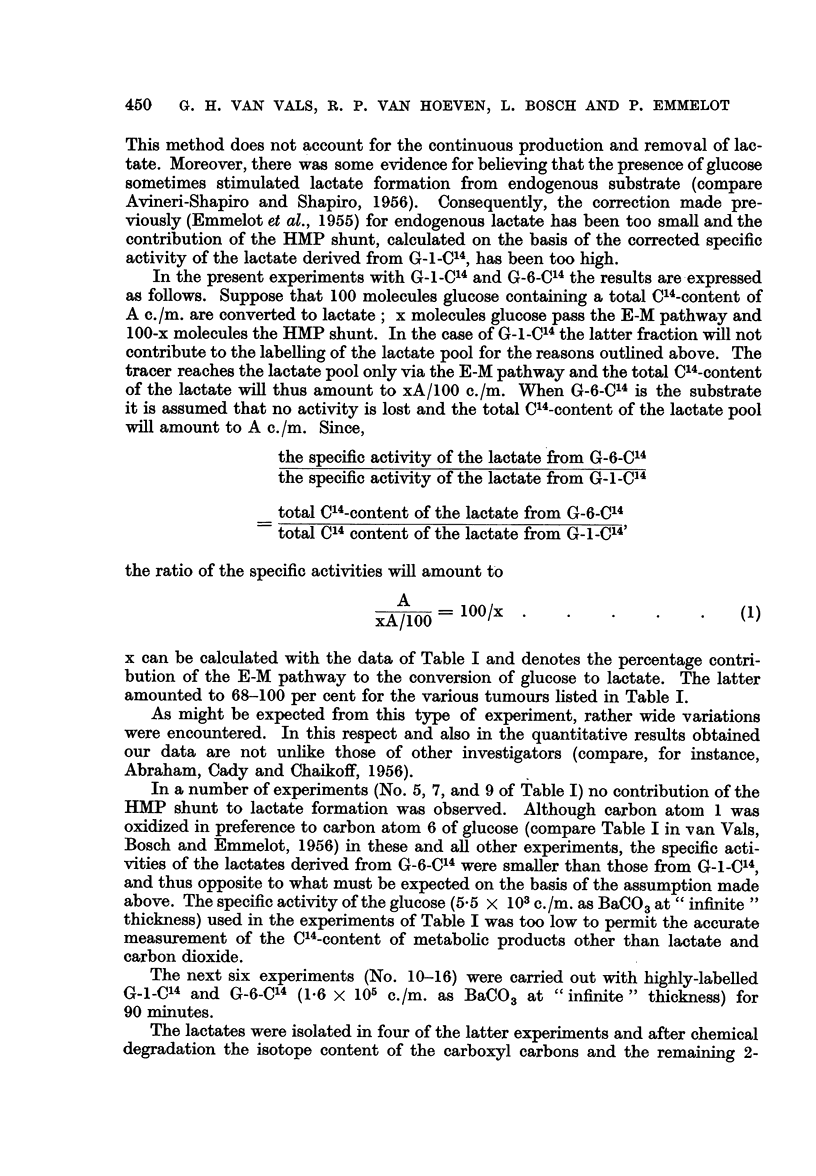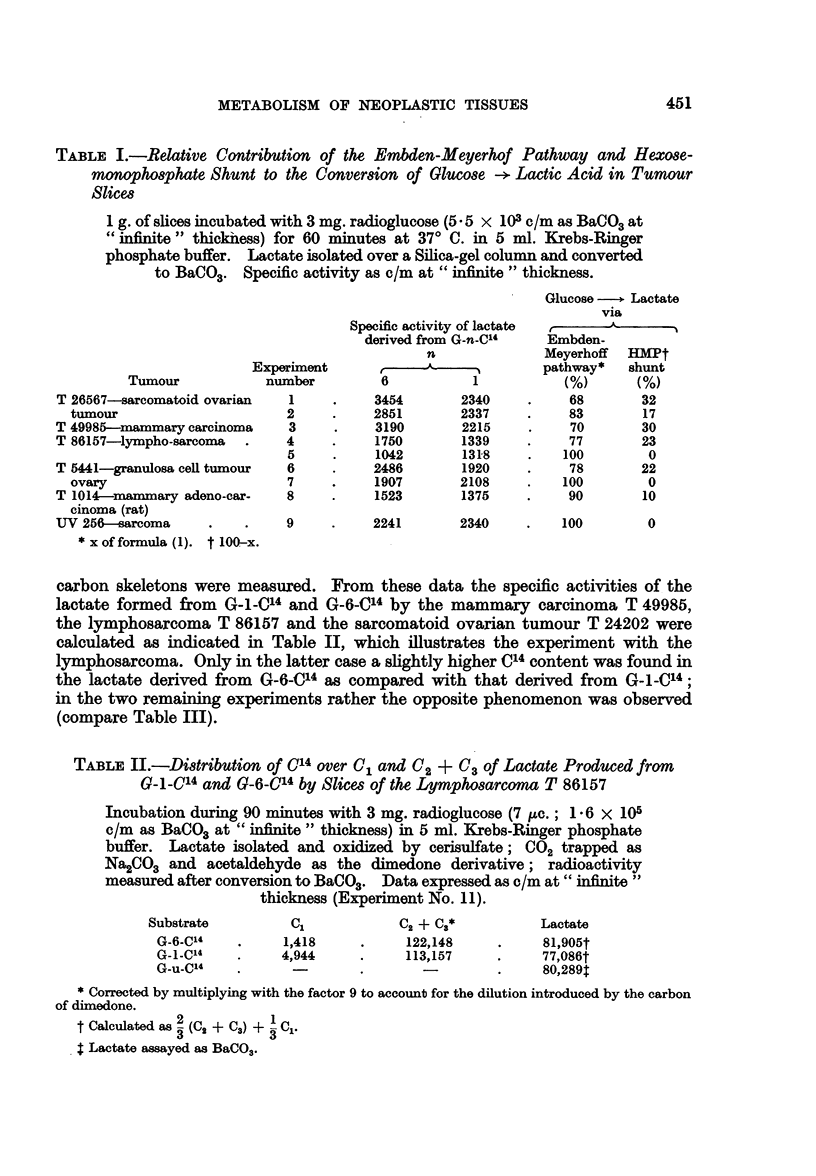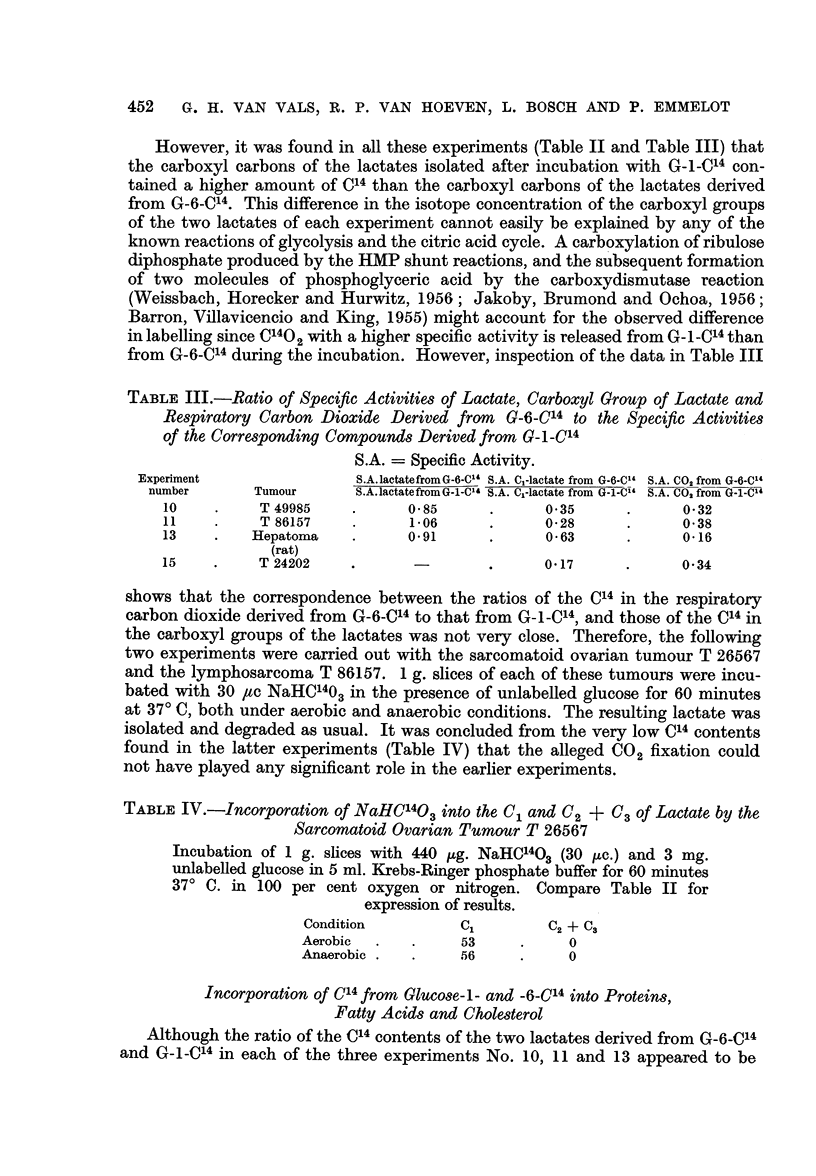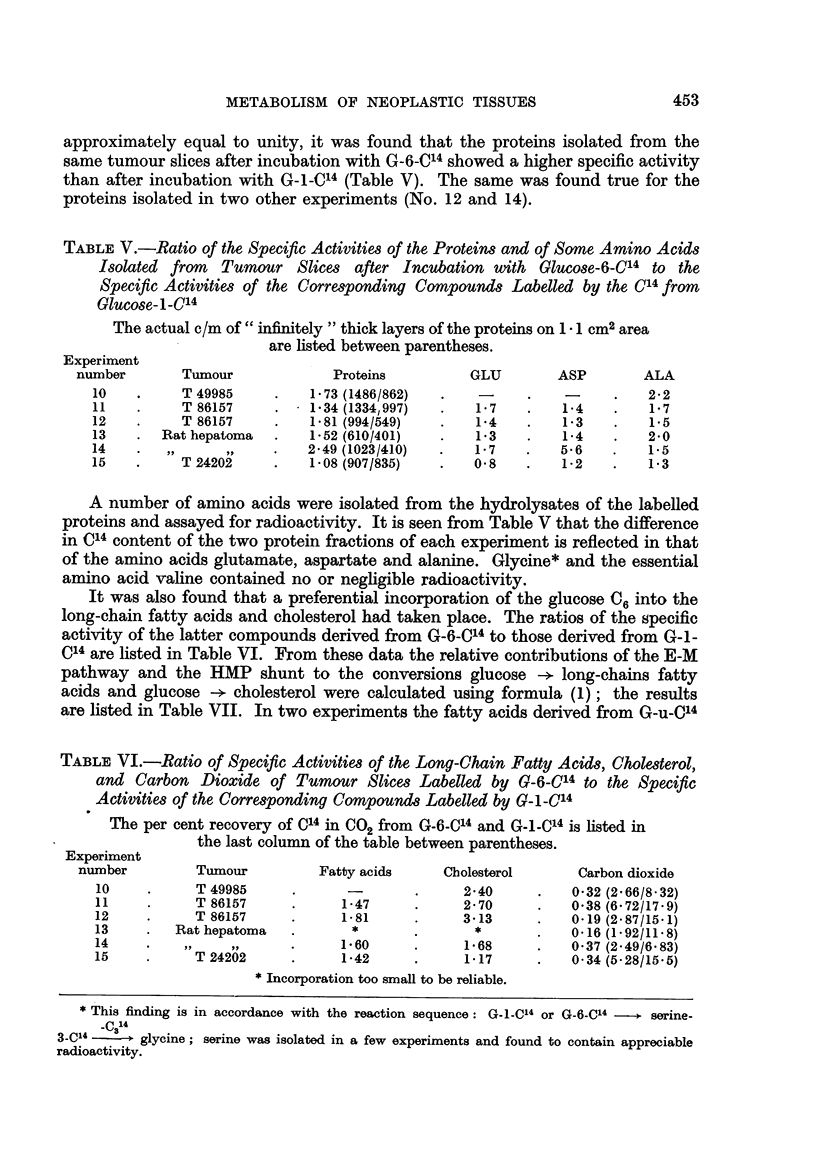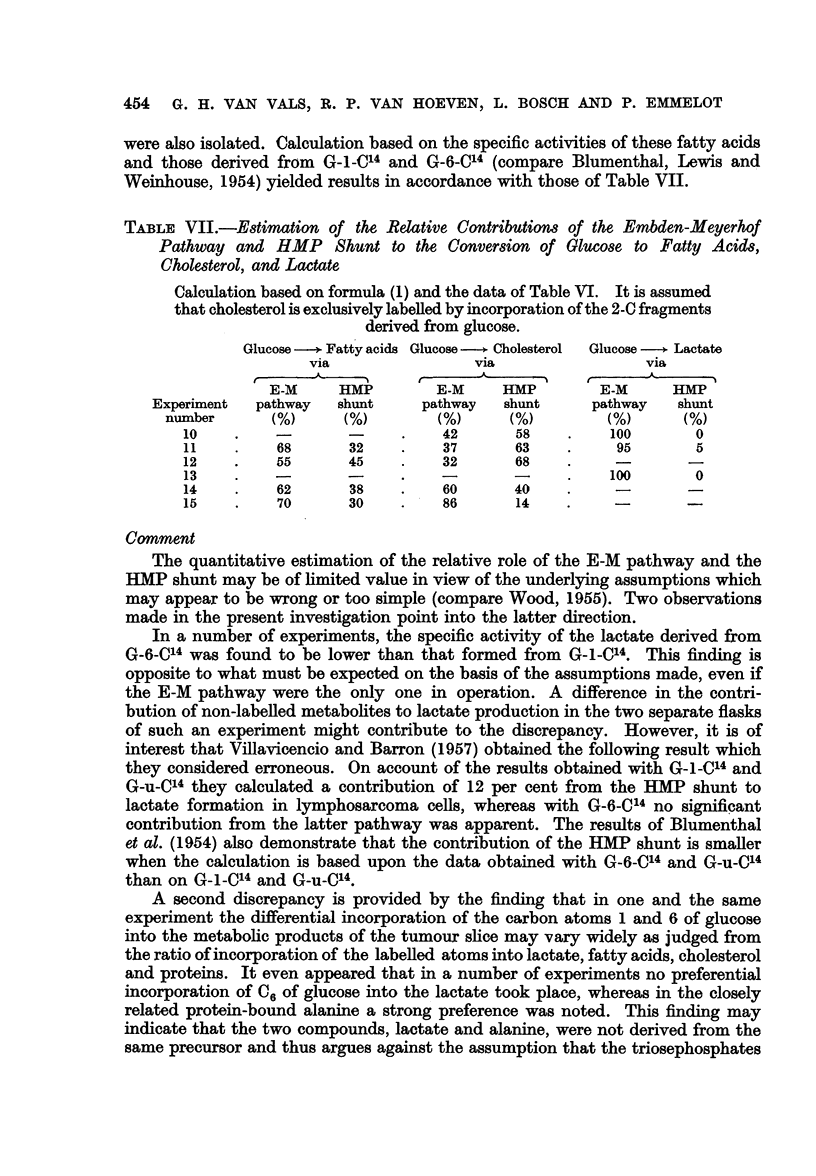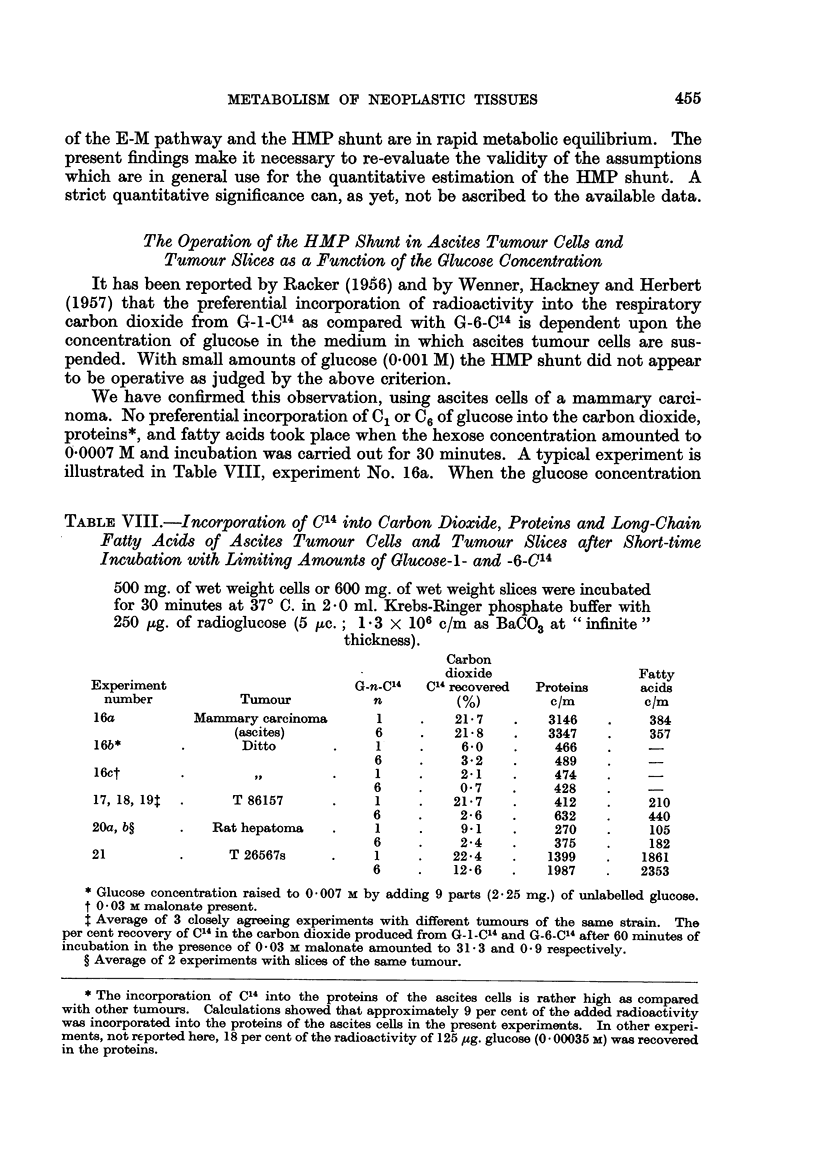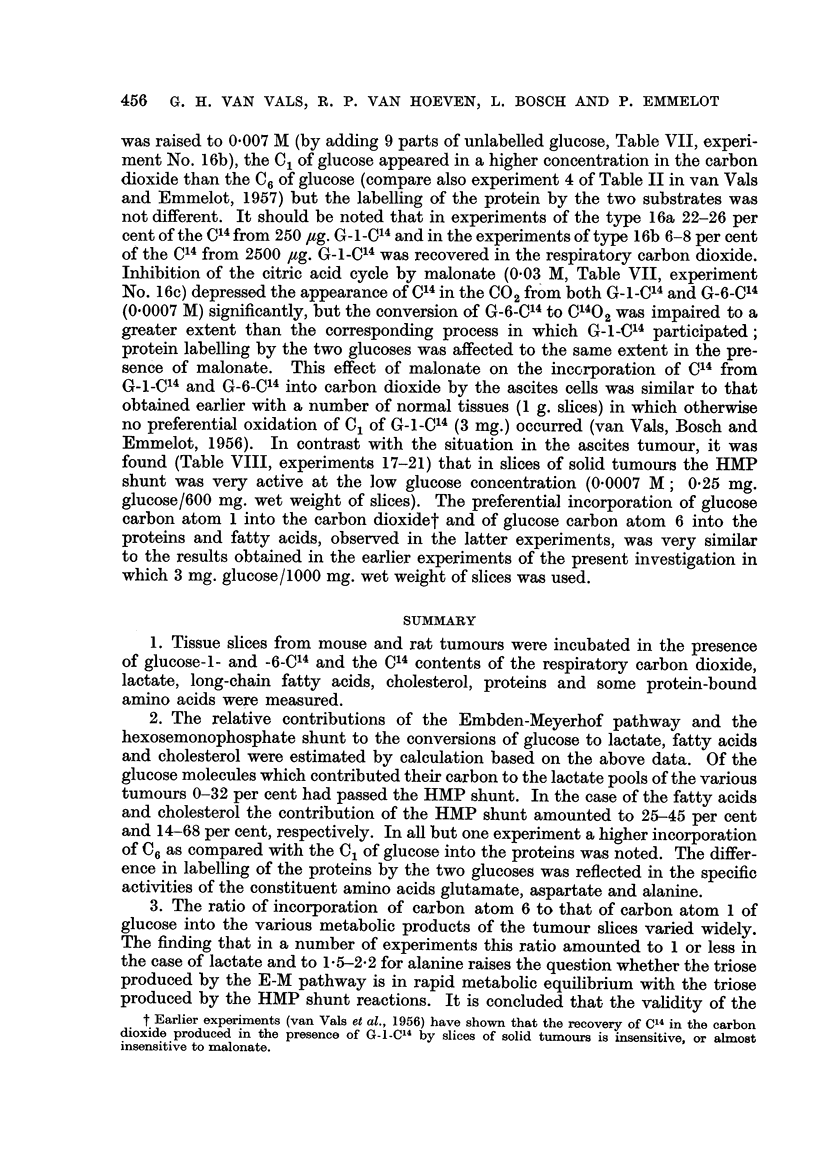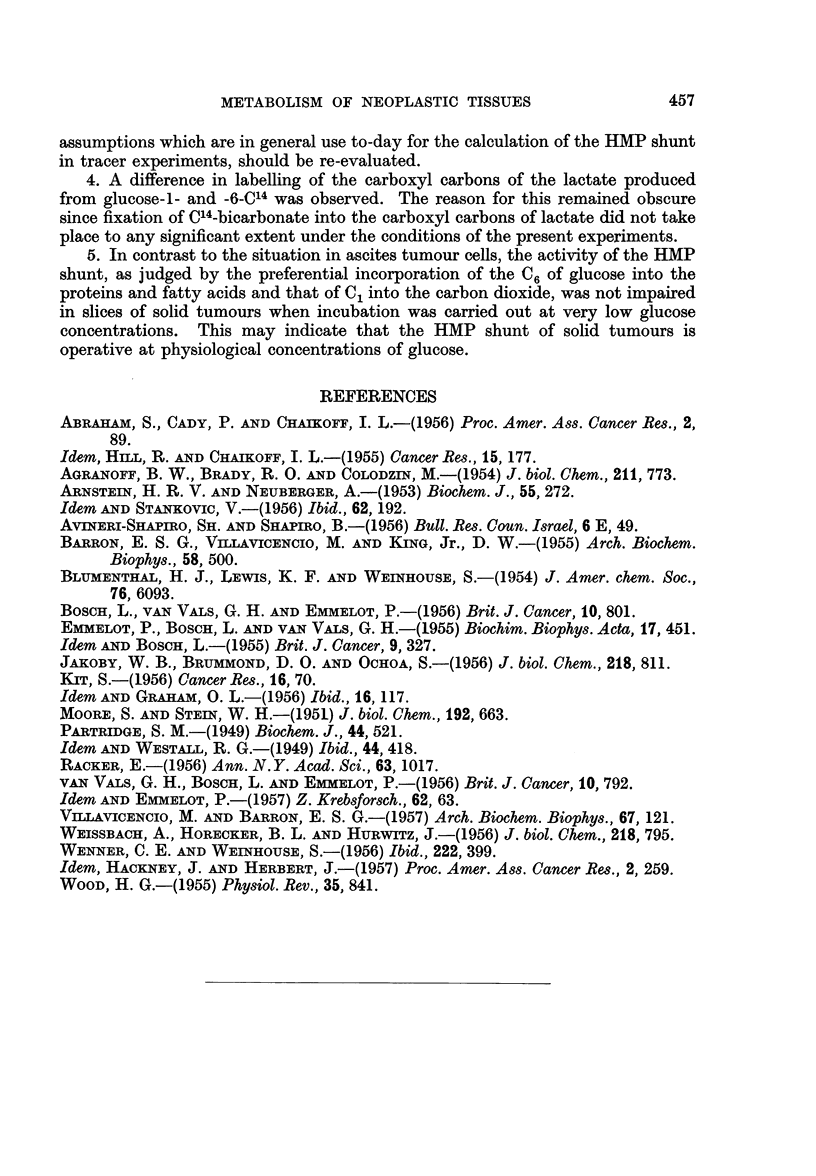# The Metabolism of Neoplastic Tissues: Further Studies on the Hexosemonophosphate Oxidative Pathway

**DOI:** 10.1038/bjc.1958.53

**Published:** 1958-09

**Authors:** G. H. van Vals, R. P. van Hoeven, L. Bosch, P. Emmelot


### THE METABOLISM OF NEOPLASTIC TISSUES : FURTHER STUDIES ON THE HEXOSEMONOPHOSPHATE OXIDATIVE PATHWAY

G. H. VAN VALS, R. P. VAN HOEVEN, L. BOSCH AND P. EMMELOT

*From the Department of Biochemistry, Antoni van Leeuwenhoek-Huis : The Netherlands Cancer Institute, Amsterdam*

Received for publication July 3, 1958

IN recent years it was recognized that glucose may be dissimilated in mammalian tissues by a route which is distinct from the classical glycolytic (Embden-Meyerhof) pathway, namely the hexosemonophosphate oxidative pathway (HMP shunt). The latter reaction sequence has been studied with several techniques, including the use of tracers in attempts to assess its quantitative significance (compare Wood, 1955).

The HMP shunt has also been shown to be operative in many neoplastic tissues of widely different origin (Agranoff, Brady and Colodzin, 1954 ; Emmelot, Bosch and van Vals, 1955 ; van Vals, Bosch and Emmelot, 1956 ; Bosch, van Vals and Emmelot, 1956 ; van Vals and Emmelot, 1957 ; Abraham, Hill and Chaikoff, 1955 ; Abraham, Cady and Chaikof, 1956 ; Wenner and Weinhouse, 1956 ; Kit, 1956 ; Kit and Graham, 1956 ; Villavicencio and Barron, 1957). In solid and ascites tumours the relative occurrence of the E-M pathway and HMP shunt has been evaluated quantitatively by measuring the differential incorporation of the carbon atoms 1 and 6 from specifically-labelled glucose into lactate or fatty acids (Wenner and Weinhouse, 1956 ; Abraham *et al.*, 1956).

In the present investigation, which was started well before the latter studies appeared in print, a similar approach was made. After a number of preliminary experiments (Emmelot *et al.*, 1955) in which lactate and carbon dioxide were isolated, we felt it necessary to check the general validity of the method by collecting information about the labelling of other cellular compounds. To this end a study was made of the incorporation of $C^{14}$ from glucose-1-$C^{14}$ and glucose-6-$C^{14}$ (G-1-$C^{14}$ and G-6-$C^{14}$, respectively) into the carbon dioxide, lactate, long-chain fatty acids, cholesterol, proteins and some protein-bound amino acids of tumour slices and ascites cells. The results of these experiments are reported below.

### MATERIAL AND METHODS

Unless stated otherwise transplanted mouse tumours were used in the present experiments. The ascites tumour (mammary carcinoma S3A obtained from Dr. G. Klein, Stockholm) was maintained in F1(Bx$C_3$H) mice. Liver tumours were induced in rats of the inbred strain R-Amsterdam by feeding p-dimethylaminoazobenzene ; a primary tumour of the mixed hepato-cholangiocellular type was used in experiment No. 13, a transplanted (third passage) tumour of the hepatocellular type in experiment No. 14 and a primary tumour of the hepatocellular type in experiment No. 20.

The incubation procedure and the isolation of the respiratory carbon dioxide, proteins, long-chain fatty acids, cholesterol and lactate, including the chemical degradation of lactate, has been described previously (Emmelot and Bosch, 1955 ; van Vals and Emmelot, 1957).

50–100 mg. protein was hydrolyzed with 6–10 ml. 6-NHCl at 100° C. for 22 hours. Glutamate and aspartate were isolated over Amberlite-IR4B and a fraction containing glycine plus alanine over Zeo-Karb 225 (Moore and Stein, 1951 ; Arnstein and Neuberger, 1953 ; Arnstein and Stankovic, 1956). An Amberlite IR-120 ($H^+$) column was used to free the latter fraction from BRY 35 and anions (Partridge, 1949 & Partridge and Westall, 1949). Glycine and alanine were separated by paper chromatography on Whatman 3 MM paper (Arnstein and Stankovic, 1956). The purity of the four amino acids was checked by paper chromatography on Whatman No. 1 paper.

Amino acids and lactate were combusted to carbon dioxide and measured as $BaCO_3$ at " infinite " thickness. Proteins, fatty acids and cholesterol digitonide were plated directly and their radioactivity was measured at " infinite " thickness, and, if necessary, correction being made for self-absorption. The radioactivity of a 1·1 cm.$^2$ area was measured under a Geiger-Müller end-window counter under our standard conditions. The c./m. at " infinite " thickness are listed as specific activity.

$NaHC^{14}O_3$ was synthesized from $BaC^{14}O_3$. G-1-$C^{14}$, G-6-$C^{14}$ and G-u-$C^{14}$ were purchased from the Radiochemical Centre (Amersham). G-6-$C^{14}$ was also obtained from the United States Atomic Energy Commission. The labelled glucoses were diluted with inactive glucose to give the desired isotope concentration.

### RESULTS AND DISCUSSION

### *The Contribution of the HMP Shunt and the Embden-Meyerhof Pathway to Lactate Formation from Glucose*

If G-1-$C^{14}$ or G-6-$C^{14}$ is converted to lactate exclusively via the E-M pathway, in which the hexose molecule is cleaved symmetrically, pyruvate-3-$C^{14}$ is produced and no radioactivity is lost. On the other hand, if a molecule of glucose enters the HMP shunt, carbon atom 1 is lost early as $CO_2$ and the carbon atoms 2 and 3 as " diose ". The resulting triose, corresponding the C-4, 5, and 6 of the original glucose, is considered to be in equilibrium with the triose derived via the E-M pathway. Thus, if G-1-$C^{14}$ serves as substrate, the HMP shunt contributes the unlabelled half of the glucose molecule to the lactate pool, whereas the labelled part of the glucose enters the lactate pool in the case of G-6-$C^{14}$. The specific activities of the lactates derived from the two labelled glucoses may then serve for the calculation of the relative contribution of the HMP shunt and E-M pathway to the conversion of glucose to lactate. This procedure, in which the dilution of the $C^{14}$ by endogenous substrates is not measured directly, has been followed in the present paper.

The dilution of the lactate pool by unlabelled lactic acid derived from endogenous substrates can be calculated by measuring the specific activity of the lactate isolated after incubation with G-u-$C^{14}$. The specific activity of the lactate derived from either G-1-$C^{14}$ or G-6-$C^{14}$ can then be corrected and used for calculating the relative occurrence of the two pathways. The latter procedure has been introduced by Blumenthal, Lewis and Weinhouse (1954). In our earlier experiments (Emmelot, Bosch and van Vals, 1955) we had sought to account for the dilution factor by measuring chemically the amount of lactate formed during incubation.

This method does not account for the continuous production and removal of lactate. Moreover, there was some evidence for believing that the presence of glucose sometimes stimulated lactate formation from endogenous substrate (compare Avineri-Shapiro and Shapiro, 1956). Consequently, the correction made previously (Emmelot *et al.*, 1955) for endogenous lactate has been too small and the contribution of the HMP shunt, calculated on the basis of the corrected specific activity of the lactate derived from G-1-$C^{14}$, has been too high.

In the present experiments with G-1-$C^{14}$ and G-6-$C^{14}$ the results are expressed as follows. Suppose that 100 molecules glucose containing a total $C^{14}$-content of A c./m. are converted to lactate ; x molecules glucose pass the E-M pathway and 100-x molecules the HMP shunt. In the case of G-1-$C^{14}$ the latter fraction will not contribute to the labelling of the lactate pool for the reasons outlined above. The tracer reaches the lactate pool only via the E-M pathway and the total $C^{14}$-content of the lactate will thus amount to xA/100 c./m. When G-6-$C^{14}$ is the substrate it is assumed that no activity is lost and the total $C^{14}$-content of the lactate pool will amount to A c./m. Since,

$$\frac{\text{the specific activity of the lactate from G-6-}C^{14}}{\text{the specific activity of the lactate from G-1-}C^{14}}$$

$$= \frac{\text{total } C^{14}\text{-content of the lactate from G-6-}C^{14}}{\text{total } C^{14} \text{ content of the lactate from G-1-}C^{14}},$$

the ratio of the specific activities will amount to

$$\frac{A}{xA/100} = 100/x \quad . \quad . \quad . \quad . \quad . \quad . \quad (1)$$

x can be calculated with the data of Table I and denotes the percentage contribution of the E-M pathway to the conversion of glucose to lactate. The latter amounted to 68–100 per cent for the various tumours listed in Table I.

As might be expected from this type of experiment, rather wide variations were encountered. In this respect and also in the quantitative results obtained our data are not unlike those of other investigators (compare, for instance, Abraham, Cady and Chaikoff, 1956).

In a number of experiments (No. 5, 7, and 9 of Table I) no contribution of the HMP shunt to lactate formation was observed. Although carbon atom 1 was oxidized in preference to carbon atom 6 of glucose (compare Table I in van Vals, Bosch and Emmelot, 1956) in these and all other experiments, the specific activities of the lactates derived from G-6-$C^{14}$ were smaller than those from G-1-$C^{14}$, and thus opposite to what must be expected on the basis of the assumption made above. The specific activity of the glucose ($5{\cdot}5 \times 10^3$ c./m. as $BaCO_3$ at " infinite " thickness) used in the experiments of Table I was too low to permit the accurate measurement of the $C^{14}$-content of metabolic products other than lactate and carbon dioxide.

The next six experiments (No. 10–16) were carried out with highly-labelled G-1-$C^{14}$ and G-6-$C^{14}$ ($1{\cdot}6 \times 10^5$ c./m. as $BaCO_3$ at " infinite " thickness) for 90 minutes.

The lactates were isolated in four of the latter experiments and after chemical degradation the isotope content of the carboxyl carbons and the remaining 2-

TABLE I.—*Relative Contribution of the Embden-Meyerhof Pathway and Hexosemonophosphate Shunt to the Conversion of Glucose → Lactic Acid in Tumour Slices*

1 g. of slices incubated with 3 mg. radioglucose ($5 \cdot 5 \times 10^3$ c/m as $BaCO_3$ at " infinite " thickness) for 60 minutes at 37° C. in 5 ml. Krebs-Ringer phosphate buffer. Lactate isolated over a Silica-gel column and converted to $BaCO_3$. Specific activity as c/m at " infinite " thickness.

| Tumour | Experiment number | Specific activity of lactate derived from G-$n$-$C^{14}$, $n$ = 6 | $n$ = 1 | Glucose → Lactate via Embden-Meyerhoff pathway* (%) | HMP† shunt (%) |
|---|---|---|---|---|---|
| T 26567—sarcomatoid ovarian | 1 | 3454 | 2340 | 68 | 32 |
| tumour | 2 | 2851 | 2337 | 83 | 17 |
| T 49985—mammary carcinoma | 3 | 3190 | 2215 | 70 | 30 |
| T 86157—lympho-sarcoma | 4 | 1750 | 1339 | 77 | 23 |
| | 5 | 1042 | 1318 | 100 | 0 |
| T 5441—granulosa cell tumour | 6 | 2486 | 1920 | 78 | 22 |
| ovary | 7 | 1907 | 2108 | 100 | 0 |
| T 1014—mammary adeno-carcinoma (rat) | 8 | 1523 | 1375 | 90 | 10 |
| UV 256—sarcoma | 9 | 2241 | 2340 | 100 | 0 |

* x of formula (1). † 100–x.

carbon skeletons were measured. From these data the specific activities of the lactate formed from G-1-$C^{14}$ and G-6-$C^{14}$ by the mammary carcinoma T 49985, the lymphosarcoma T 86157 and the sarcomatoid ovarian tumour T 24202 were calculated as indicated in Table II, which illustrates the experiment with the lymphosarcoma. Only in the latter case a slightly higher $C^{14}$ content was found in the lactate derived from G-6-$C^{14}$ as compared with that derived from G-1-$C^{14}$; in the two remaining experiments rather the opposite phenomenon was observed (compare Table III).

TABLE II.—*Distribution of $C^{14}$ over $C_1$ and $C_2 + C_3$ of Lactate Produced from G-1-$C^{14}$ and G-6-$C^{14}$ by Slices of the Lymphosarcoma T* 86157

Incubation during 90 minutes with 3 mg. radioglucose (7 μc.; $1 \cdot 6 \times 10^5$ c/m as $BaCO_3$ at " infinite " thickness) in 5 ml. Krebs-Ringer phosphate buffer. Lactate isolated and oxidized by cerisulfate; $CO_2$ trapped as $Na_2CO_3$ and acetaldehyde as the dimedone derivative; radioactivity measured after conversion to $BaCO_3$. Data expressed as c/m at " infinite " thickness (Experiment No. 11).

| Substrate | $C_1$ | $C_2 + C_3$* | Lactate |
|---|---|---|---|
| G-6-$C^{14}$ | 1,418 | 122,148 | 81,905† |
| G-1-$C^{14}$ | 4,944 | 113,157 | 77,086† |
| G-u-$C^{14}$ | — | — | 80,289‡ |

* Corrected by multiplying with the factor 9 to account for the dilution introduced by the carbon of dimedone.

† Calculated as $\frac{2}{3}(C_2 + C_3) + \frac{1}{3}C_1$.

‡ Lactate assayed as $BaCO_3$.

However, it was found in all these experiments (Table II and Table III) that the carboxyl carbons of the lactates isolated after incubation with G-1-$C^{14}$ contained a higher amount of $C^{14}$ than the carboxyl carbons of the lactates derived from G-6-$C^{14}$. This difference in the isotope concentration of the carboxyl groups of the two lactates of each experiment cannot easily be explained by any of the known reactions of glycolysis and the citric acid cycle. A carboxylation of ribulose diphosphate produced by the HMP shunt reactions, and the subsequent formation of two molecules of phosphoglyceric acid by the carboxydismutase reaction (Weissbach, Horecker and Hurwitz, 1956; Jakoby, Brumond and Ochoa, 1956; Barron, Villavicencio and King, 1955) might account for the observed difference in labelling since $C^{14}O_2$ with a higher specific activity is released from G-1-$C^{14}$ than from G-6-$C^{14}$ during the incubation. However, inspection of the data in Table III

TABLE III.—*Ratio of Specific Activities of Lactate, Carboxyl Group of Lactate and Respiratory Carbon Dioxide Derived from G-6-$C^{14}$ to the Specific Activities of the Corresponding Compounds Derived from G-1-$C^{14}$*

S.A. = Specific Activity.

| Experiment number | Tumour | S.A. lactate from G-6-$C^{14}$ / S.A. lactate from G-1-$C^{14}$ | S.A. $C_1$-lactate from G-6-$C^{14}$ / S.A. $C_1$-lactate from G-1-$C^{14}$ | S.A. $CO_2$ from G-6-$C^{14}$ / S.A. $CO_2$ from G-1-$C^{14}$ |
|---|---|---|---|---|
| 10 | T 49985 | 0·85 | 0·35 | 0·32 |
| 11 | T 86157 | 1·06 | 0·28 | 0·38 |
| 13 | Hepatoma (rat) | 0·91 | 0·63 | 0·16 |
| 15 | T 24202 | — | 0·17 | 0·34 |

shows that the correspondence between the ratios of the $C^{14}$ in the respiratory carbon dioxide derived from G-6-$C^{14}$ to that from G-1-$C^{14}$, and those of the $C^{14}$ in the carboxyl groups of the lactates was not very close. Therefore, the following two experiments were carried out with the sarcomatoid ovarian tumour T 26567 and the lymphosarcoma T 86157. 1 g. slices of each of these tumours were incubated with 30 $\mu c$ $NaHC^{14}O_3$ in the presence of unlabelled glucose for 60 minutes at 37° C, both under aerobic and anaerobic conditions. The resulting lactate was isolated and degraded as usual. It was concluded from the very low $C^{14}$ contents found in the latter experiments (Table IV) that the alleged $CO_2$ fixation could not have played any significant role in the earlier experiments.

TABLE IV.—*Incorporation of $NaHC^{14}O_3$ into the $C_1$ and $C_2 + C_3$ of Lactate by the Sarcomatoid Ovarian Tumour T 26567*

Incubation of 1 g. slices with 440 $\mu$g. $NaHC^{14}O_3$ (30 $\mu$c.) and 3 mg. unlabelled glucose in 5 ml. Krebs-Ringer phosphate buffer for 60 minutes 37° C. in 100 per cent oxygen or nitrogen. Compare Table II for expression of results.

| Condition | $C_1$ | $C_2 + C_3$ |
|---|---|---|
| Aerobic | 53 | 0 |
| Anaerobic | 56 | 0 |

### *Incorporation of $C^{14}$ from Glucose-1- and -6-$C^{14}$ into Proteins, Fatty Acids and Cholesterol*

Although the ratio of the $C^{14}$ contents of the two lactates derived from G-6-$C^{14}$ and G-1-$C^{14}$ in each of the three experiments No. 10, 11 and 13 appeared to be approximately equal to unity, it was found that the proteins isolated from the same tumour slices after incubation with G-6-$C^{14}$ showed a higher specific activity than after incubation with G-1-$C^{14}$ (Table V). The same was found true for the proteins isolated in two other experiments (No. 12 and 14).

TABLE V.—*Ratio of the Specific Activities of the Proteins and of Some Amino Acids Isolated from Tumour Slices after Incubation with Glucose-6-$C^{14}$ to the Specific Activities of the Corresponding Compounds Labelled by the $C^{14}$ from Glucose-1-$C^{14}$*

The actual c/m of " infinitely " thick layers of the proteins on 1·1 $cm^2$ area are listed between parentheses.

| Experiment number | Tumour | Proteins | GLU | ASP | ALA |
|---|---|---|---|---|---|
| 10 | T 49985 | 1·73 (1486/862) | — | — | 2·2 |
| 11 | T 86157 | 1·34 (1334/997) | 1·7 | 1·4 | 1·7 |
| 12 | T 86157 | 1·81 (994/549) | 1·4 | 1·3 | 1·5 |
| 13 | Rat hepatoma | 1·52 (610/401) | 1·3 | 1·4 | 2·0 |
| 14 | „ „ | 2·49 (1023/410) | 1·7 | 5·6 | 1·5 |
| 15 | T 24202 | 1·08 (907/835) | 0·8 | 1·2 | 1·3 |

A number of amino acids were isolated from the hydrolysates of the labelled proteins and assayed for radioactivity. It is seen from Table V that the difference in $C^{14}$ content of the two protein fractions of each experiment is reflected in that of the amino acids glutamate, aspartate and alanine. Glycine* and the essential amino acid valine contained no or negligible radioactivity.

It was also found that a preferential incorporation of the glucose $C_6$ into the long-chain fatty acids and cholesterol had taken place. The ratios of the specific activity of the latter compounds derived from G-6-$C^{14}$ to those derived from G-1-$C^{14}$ are listed in Table VI. From these data the relative contributions of the E-M pathway and the HMP shunt to the conversions glucose → long-chains fatty acids and glucose → cholesterol were calculated using formula (1) ; the results are listed in Table VII. In two experiments the fatty acids derived from G-u-$C^{14}$

TABLE VI.—*Ratio of Specific Activities of the Long-Chain Fatty Acids, Cholesterol, and Carbon Dioxide of Tumour Slices Labelled by G-6-$C^{14}$ to the Specific Activities of the Corresponding Compounds Labelled by G-1-$C^{14}$*

The per cent recovery of $C^{14}$ in $CO_2$ from G-6-$C^{14}$ and G-1-$C^{14}$ is listed in the last column of the table between parentheses.

| Experiment number | Tumour | Fatty acids | Cholesterol | Carbon dioxide |
|---|---|---|---|---|
| 10 | T 49985 | — | 2·40 | 0·32 (2·66/8·32) |
| 11 | T 86157 | 1·47 | 2·70 | 0·38 (6·72/17·9) |
| 12 | T 86157 | 1·81 | 3·13 | 0·19 (2·87/15·1) |
| 13 | Rat hepatoma | * | * | 0·16 (1·92/11·8) |
| 14 | „ „ | 1·60 | 1·68 | 0·37 (2·49/6·83) |
| 15 | T 24202 | 1·42 | 1·17 | 0·34 (5·28/15·5) |

* Incorporation too small to be reliable.

* This finding is in accordance with the reaction sequence : G-1-$C^{14}$ or G-6-$C^{14}$ $\longrightarrow$ serine-3-$C^{14}$ $\xrightarrow{-C_3^{14}}$ glycine ; serine was isolated in a few experiments and found to contain appreciable radioactivity.

were also isolated. Calculation based on the specific activities of these fatty acids and those derived from G-1-$C^{14}$ and G-6-$C^{14}$ (compare Blumenthal, Lewis and Weinhouse, 1954) yielded results in accordance with those of Table VII.

TABLE VII.—*Estimation of the Relative Contributions of the Embden-Meyerhof Pathway and HMP Shunt to the Conversion of Glucose to Fatty Acids, Cholesterol, and Lactate*

Calculation based on formula (1) and the data of Table VI. It is assumed that cholesterol is exclusively labelled by incorporation of the 2-C fragments derived from glucose.

| | Glucose ⟶ Fatty acids via | | Glucose ⟶ Cholesterol via | | Glucose ⟶ Lactate via | |
|---|---|---|---|---|---|---|
| Experiment number | E-M pathway (%) | HMP shunt (%) | E-M pathway (%) | HMP shunt (%) | E-M pathway (%) | HMP shunt (%) |
| 10 | — | — | 42 | 58 | 100 | 0 |
| 11 | 68 | 32 | 37 | 63 | 95 | 5 |
| 12 | 55 | 45 | 32 | 68 | — | — |
| 13 | — | — | — | — | 100 | 0 |
| 14 | 62 | 38 | 60 | 40 | — | — |
| 15 | 70 | 30 | 86 | 14 | — | — |

*Comment*

The quantitative estimation of the relative role of the E-M pathway and the HMP shunt may be of limited value in view of the underlying assumptions which may appear to be wrong or too simple (compare Wood, 1955). Two observations made in the present investigation point into the latter direction.

In a number of experiments, the specific activity of the lactate derived from G-6-$C^{14}$ was found to be lower than that formed from G-1-$C^{14}$. This finding is opposite to what must be expected on the basis of the assumptions made, even if the E-M pathway were the only one in operation. A difference in the contribution of non-labelled metabolites to lactate production in the two separate flasks of such an experiment might contribute to the discrepancy. However, it is of interest that Villavicencio and Barron (1957) obtained the following result which they considered erroneous. On account of the results obtained with G-1-$C^{14}$ and G-u-$C^{14}$ they calculated a contribution of 12 per cent from the HMP shunt to lactate formation in lymphosarcoma cells, whereas with G-6-$C^{14}$ no significant contribution from the latter pathway was apparent. The results of Blumenthal *et al.* (1954) also demonstrate that the contribution of the HMP shunt is smaller when the calculation is based upon the data obtained with G-6-$C^{14}$ and G-u-$C^{14}$ than on G-1-$C^{14}$ and G-u-$C^{14}$.

A second discrepancy is provided by the finding that in one and the same experiment the differential incorporation of the carbon atoms 1 and 6 of glucose into the metabolic products of the tumour slice may vary widely as judged from the ratio of incorporation of the labelled atoms into lactate, fatty acids, cholesterol and proteins. It even appeared that in a number of experiments no preferential incorporation of $C_6$ of glucose into the lactate took place, whereas in the closely related protein-bound alanine a strong preference was noted. This finding may indicate that the two compounds, lactate and alanine, were not derived from the same precursor and thus argues against the assumption that the triosephosphates of the E-M pathway and the HMP shunt are in rapid metabolic equilibrium. The present findings make it necessary to re-evaluate the validity of the assumptions which are in general use for the quantitative estimation of the HMP shunt. A strict quantitative significance can, as yet, not be ascribed to the available data.

### *The Operation of the HMP Shunt in Ascites Tumour Cells and Tumour Slices as a Function of the Glucose Concentration*

It has been reported by Racker (1956) and by Wenner, Hackney and Herbert (1957) that the preferential incorporation of radioactivity into the respiratory carbon dioxide from G-1-$C^{14}$ as compared with G-6-$C^{14}$ is dependent upon the concentration of glucose in the medium in which ascites tumour cells are suspended. With small amounts of glucose (0·001 M) the HMP shunt did not appear to be operative as judged by the above criterion.

We have confirmed this observation, using ascites cells of a mammary carcinoma. No preferential incorporation of $C_1$ or $C_6$ of glucose into the carbon dioxide, proteins*, and fatty acids took place when the hexose concentration amounted to 0·0007 M and incubation was carried out for 30 minutes. A typical experiment is illustrated in Table VIII, experiment No. 16a. When the glucose concentration

TABLE VIII.—*Incorporation of $C^{14}$ into Carbon Dioxide, Proteins and Long-Chain Fatty Acids of Ascites Tumour Cells and Tumour Slices after Short-time Incubation with Limiting Amounts of Glucose-1- and -6-$C^{14}$*

500 mg. of wet weight cells or 600 mg. of wet weight slices were incubated for 30 minutes at 37° C. in 2·0 ml. Krebs-Ringer phosphate buffer with 250 $\mu$g. of radioglucose (5 $\mu$c.; $1·3 \times 10^6$ c/m as $BaCO_3$ at "infinite" thickness).

| Experiment number | Tumour | G-*n*-$C^{14}$ *n* | Carbon dioxide $C^{14}$ recovered (%) | Proteins c/m | Fatty acids c/m |
|---|---|---|---|---|---|
| 16*a* | Mammary carcinoma | 1 | 21·7 | 3146 | 384 |
| | (ascites) | 6 | 21·8 | 3347 | 357 |
| 16*b** | Ditto | 1 | 6·0 | 466 | — |
| | | 6 | 3·2 | 489 | — |
| 16*c*† | ,, | 1 | 2·1 | 474 | — |
| | | 6 | 0·7 | 428 | — |
| 17, 18, 19‡ | T 86157 | 1 | 21·7 | 412 | 210 |
| | | 6 | 2·6 | 632 | 440 |
| 20*a*, *b*§ | Rat hepatoma | 1 | 9·1 | 270 | 105 |
| | | 6 | 2·4 | 375 | 182 |
| 21 | T 26567s | 1 | 22·4 | 1399 | 1861 |
| | | 6 | 12·6 | 1987 | 2353 |

* Glucose concentration raised to 0·007 M by adding 9 parts (2·25 mg.) of unlabelled glucose.
† 0·03 M malonate present.
‡ Average of 3 closely agreeing experiments with different tumours of the same strain. The per cent recovery of $C^{14}$ in the carbon dioxide produced from G-1-$C^{14}$ and G-6-$C^{14}$ after 60 minutes of incubation in the presence of 0·03 M malonate amounted to 31·3 and 0·9 respectively.
§ Average of 2 experiments with slices of the same tumour.

* The incorporation of $C^{14}$ into the proteins of the ascites cells is rather high as compared with other tumours. Calculations showed that approximately 9 per cent of the added radioactivity was incorporated into the proteins of the ascites cells in the present experiments. In other experiments, not reported here, 18 per cent of the radioactivity of 125 $\mu$g. glucose (0·00035 M) was recovered in the proteins.

was raised to 0·007 M (by adding 9 parts of unlabelled glucose, Table VII, experiment No. 16b), the $C_1$ of glucose appeared in a higher concentration in the carbon dioxide than the $C_6$ of glucose (compare also experiment 4 of Table II in van Vals and Emmelot, 1957) but the labelling of the protein by the two substrates was not different. It should be noted that in experiments of the type 16a 22–26 per cent of the $C^{14}$ from 250 $\mu$g. G-1-$C^{14}$ and in the experiments of type 16b 6–8 per cent of the $C^{14}$ from 2500 $\mu$g. G-1-$C^{14}$ was recovered in the respiratory carbon dioxide. Inhibition of the citric acid cycle by malonate (0·03 M, Table VII, experiment No. 16c) depressed the appearance of $C^{14}$ in the $CO_2$ from both G-1-$C^{14}$ and G-6-$C^{14}$ (0·0007 M) significantly, but the conversion of G-6-$C^{14}$ to $C^{14}O_2$ was impaired to a greater extent than the corresponding process in which G-1-$C^{14}$ participated; protein labelling by the two glucoses was affected to the same extent in the presence of malonate. This effect of malonate on the incorporation of $C^{14}$ from G-1-$C^{14}$ and G-6-$C^{14}$ into carbon dioxide by the ascites cells was similar to that obtained earlier with a number of normal tissues (1 g. slices) in which otherwise no preferential oxidation of $C_1$ of G-1-$C^{14}$ (3 mg.) occurred (van Vals, Bosch and Emmelot, 1956). In contrast with the situation in the ascites tumour, it was found (Table VIII, experiments 17–21) that in slices of solid tumours the HMP shunt was very active at the low glucose concentration (0·0007 M; 0·25 mg. glucose/600 mg. wet weight of slices). The preferential incorporation of glucose carbon atom 1 into the carbon dioxide† and of glucose carbon atom 6 into the proteins and fatty acids, observed in the latter experiments, was very similar to the results obtained in the earlier experiments of the present investigation in which 3 mg. glucose/1000 mg. wet weight of slices was used.

SUMMARY

1. Tissue slices from mouse and rat tumours were incubated in the presence of glucose-1- and -6-$C^{14}$ and the $C^{14}$ contents of the respiratory carbon dioxide, lactate, long-chain fatty acids, cholesterol, proteins and some protein-bound amino acids were measured.

2. The relative contributions of the Embden-Meyerhof pathway and the hexosemonophosphate shunt to the conversions of glucose to lactate, fatty acids and cholesterol were estimated by calculation based on the above data. Of the glucose molecules which contributed their carbon to the lactate pools of the various tumours 0–32 per cent had passed the HMP shunt. In the case of the fatty acids and cholesterol the contribution of the HMP shunt amounted to 25–45 per cent and 14–68 per cent, respectively. In all but one experiment a higher incorporation of $C_6$ as compared with the $C_1$ of glucose into the proteins was noted. The difference in labelling of the proteins by the two glucoses was reflected in the specific activities of the constituent amino acids glutamate, aspartate and alanine.

3. The ratio of incorporation of carbon atom 6 to that of carbon atom 1 of glucose into the various metabolic products of the tumour slices varied widely. The finding that in a number of experiments this ratio amounted to 1 or less in the case of lactate and to 1·5–2·2 for alanine raises the question whether the triose produced by the E-M pathway is in rapid metabolic equilibrium with the triose produced by the HMP shunt reactions. It is concluded that the validity of the

† Earlier experiments (van Vals *et al.*, 1956) have shown that the recovery of $C^{14}$ in the carbon dioxide produced in the presence of G-1-$C^{14}$ by slices of solid tumours is insensitive, or almost insensitive to malonate.

assumptions which are in general use to-day for the calculation of the HMP shunt in tracer experiments, should be re-evaluated.

4. A difference in labelling of the carboxyl carbons of the lactate produced from glucose-1- and -6-$C^{14}$ was observed. The reason for this remained obscure since fixation of $C^{14}$-bicarbonate into the carboxyl carbons of lactate did not take place to any significant extent under the conditions of the present experiments.

5. In contrast to the situation in ascites tumour cells, the activity of the HMP shunt, as judged by the preferential incorporation of the $C_6$ of glucose into the proteins and fatty acids and that of $C_1$ into the carbon dioxide, was not impaired in slices of solid tumours when incubation was carried out at very low glucose concentrations. This may indicate that the HMP shunt of solid tumours is operative at physiological concentrations of glucose.

### REFERENCES

Abraham, S., Cady, P. and Chaikoff, I. L.—(1956) *Proc. Amer. Ass. Cancer Res.*, **2**, 89.

*Idem*, Hill, R. and Chaikoff, I. L.—(1955) *Cancer Res.*, **15**, 177.

Agranoff, B. W., Brady, R. O. and Colodzin, M.—(1954) *J. biol. Chem.*, **211**, 773.

Arnstein, H. R. V. and Neuberger, A.—(1953) *Biochem. J.*, **55**, 272.

*Idem* and Stankovic, V.—(1956) *Ibid.*, **62**, 192.

Avineri-Shapiro, Sh. and Shapiro, B.—(1956) *Bull. Res. Coun. Israel*, **6** E, 49.

Barron, E. S. G., Villavicencio, M. and King, Jr., D. W.—(1955) *Arch. Biochem. Biophys.*, **58**, 500.

Blumenthal, H. J., Lewis, K. F. and Weinhouse, S.—(1954) *J. Amer. chem. Soc.*, **76**, 6093.

Bosch, L., van Vals, G. H. and Emmelot, P.—(1956) *Brit. J. Cancer*, **10**, 801.

Emmelot, P., Bosch, L. and van Vals, G. H.—(1955) *Biochim. Biophys. Acta*, **17**, 451.

*Idem* and Bosch, L.—(1955) *Brit. J. Cancer*, **9**, 327.

Jakoby, W. B., Brummond, D. O. and Ochoa, S.—(1956) *J. biol. Chem.*, **218**, 811.

Kit, S.—(1956) *Cancer Res.*, **16**, 70.

*Idem* and Graham, O. L.—(1956) *Ibid.*, **16**, 117.

Moore, S. and Stein, W. H.—(1951) *J. biol. Chem.*, **192**, 663.

Partridge, S. M.—(1949) *Biochem. J.*, **44**, 521.

*Idem* and Westall, R. G.—(1949) *Ibid.*, **44**, 418.

Racker, E.—(1956) *Ann. N.Y. Acad. Sci.*, **63**, 1017.

van Vals, G. H., Bosch, L. and Emmelot, P.—(1956) *Brit. J. Cancer*, **10**, 792.

*Idem* and Emmelot, P.—(1957) *Z. Krebsforsch.*, **62**, 63.

Villavicencio, M. and Barron, E. S. G.—(1957) *Arch. Biochem. Biophys.*, **67**, 121.

Weissbach, A., Horecker, B. L. and Hurwitz, J.—(1956) *J. biol. Chem.*, **218**, 795.

Wenner, C. E. and Weinhouse, S.—(1956) *Ibid.*, **222**, 399.

*Idem*, Hackney, J. and Herbert, J.—(1957) *Proc. Amer. Ass. Cancer Res.*, **2**, 259.

Wood, H. G.—(1955) *Physiol. Rev.*, **35**, 841.